# Anti-Inflammatory Effect of Licochalcone A via Regulation of ORAI1 and K^+^ Channels in T-Lymphocytes

**DOI:** 10.3390/ijms221910847

**Published:** 2021-10-07

**Authors:** Hong T. L. Phan, Hyun J. Kim, Sungwoo Jo, Woo K. Kim, Wan Namkung, Joo H. Nam

**Affiliations:** 1 Department of Physiology, Dongguk University College of Medicine, 123 Dongdae-ro, Gyeongju 38066, Korea; phanlamhong.hatinh@gmail.com; 2 Channelopathy Research Center (CRC), Dongguk University College of Medicine, 32 Dongguk-ro, Goyang 10326, Korea; designed_hj@naver.com (H.J.K.); wk2kim@naver.com (W.K.K.); 3 Yonsei Institute of Pharmaceutical Sciences, College of Pharmacy, Yonsei University, 85 Songdogwahak-ro, Incheon 21983, Korea; ferinus@gmail.com; 4 Department of Internal Medicine Graduate School of Medicine, Dongguk University, 27 Dongguk-ro, Goyang 10326, Korea

**Keywords:** licochalcone A, store-operated calcium entry, calcium-release-activated calcium channel protein 1, calcium-activated potassium channel, voltage-gated potassium channel, anti-inflammatory effect

## Abstract

Calcium signaling plays a vital role in the regulation of various cellular processes, including activation, proliferation, and differentiation of T-lymphocytes, which is mediated by ORAI1 and potassium (K^+^) channels. These channels have also been identified as highly attractive therapeutic targets for immune-related diseases. Licochalcone A is a licorice-derived chalconoid known for its multifaceted beneficial effects in pharmacological treatments, including its anti-inflammatory, anti-asthmatic, antioxidant, antimicrobial, and antitumorigenic properties. However, its anti-inflammatory effects involving ion channels in lymphocytes remain unclear. Thus, the present study aimed to investigate whether licochalcone A inhibits ORAI1 and K^+^ channels in T-lymphocytes. Our results indicated that licochalcone A suppressed all three channels (ORAI1, Kv1.3, and KCa3.1) in a concentration-dependent matter, with IC_50_ values of 2.97 ± 1.217 µM, 0.83 ± 1.222 µM, and 11.21 ± 1.07 µM, respectively. Of note, licochalcone A exerted its suppressive effects on the IL-2 secretion and proliferation in CD3 and CD28 antibody-induced T-cells. These results indicate that the use of licochalcone A may provide an effective treatment strategy for inflammation-related immune diseases.

## 1. Introduction

Calcium signaling is of fundamental importance in the regulation of physiological events, such as activation, proliferation, and differentiation in a variety of immune cells, including T-lymphocytes [1,2]. Under resting state, the intracellular calcium concentration ([Ca^2+^]_i_) of T-lymphocytes is approximately 100 nM, which transiently elevates to 1 µM when surface receptors of T-cells are stimulated by the complexes of antigen and major histocompatibility complex; this triggers the activation state of phospholipase C-gamma, which subsequently cleaves phosphatidylinositol-4,5-bisphosphate into two signaling molecules, including inositol-1,4,5-trisphosphate (IP_3_) [2,3,4]. The release of IP_3_ results in rapid increases of ([Ca^2+^]_i_) from the endoplasmic reticulum (ER) and outside the cell [3]. In particular, IP_3_ interacts with its receptor located in the ER, causing the movement of Ca^2+^ from the ER to the cytoplasm, consequently depleting the Ca^2+^ stores that would enable stromal interaction molecular 1 (STIM1) to redistribute and activate the ORAI1 channels, a vital subunit of calcium-release-activated calcium channels. In turn, it promotes the entry of Ca^2+^ across the plasma membrane, known as store-operated Ca^2+^ entry (SOCE) [1,5,6]. The increase in ([Ca^2+^]_i_) results in the augmented transcription of the nuclear factor of activated T-cells, which triggers cytokine release, including IL-2, and T-cell proliferation. In addition to the ORAI1 channel, the calcium-activated KCa3.1 and the voltage-gated Kv1.3 are fundamental to the maintenance of the negative resting membrane potential that influences the driving force for the influx of Ca^2+^ via ORAI1 channels in lymphocytes [6,7,8,9]. Therefore, inhibitors of all three channels might provide potential therapeutic strategies for inflammatory-associated diseases.

Licochalcone A, a bioactive chalcone constituent extracted from the licorice roots of the *Glycyrrhiza* species, composed of *G. uralensis*, *G. pallidiflora*, *G. inflata*, and *G. glabra* [10,11], has been well identified to possess anti-inflammatory, anti-asthmatic, antioxidant, antimicrobial, and antitumorigenic properties [12,13,14,15,16,17]. It is important to note that the anti-inflammatory activity of licochalcone A has been previously reported in numerous studies [10,14,16,17,18,19,20,21,22,23]. However, the ion channel-based mechanism underlying its anti-inflammatory effect remains elusive. Therefore, the present study aimed to identify the effect of licochalcone A via suppression of ORAI1, Kv1.3, and KCa3.1, and the related physiological effects.

## 2. Results

### 2.1. Licochalcone A Inhibited ORAI1 Currents in HEK293T Cells Coexpressing hORAI1 with hSTIM1

**Figure 1:**
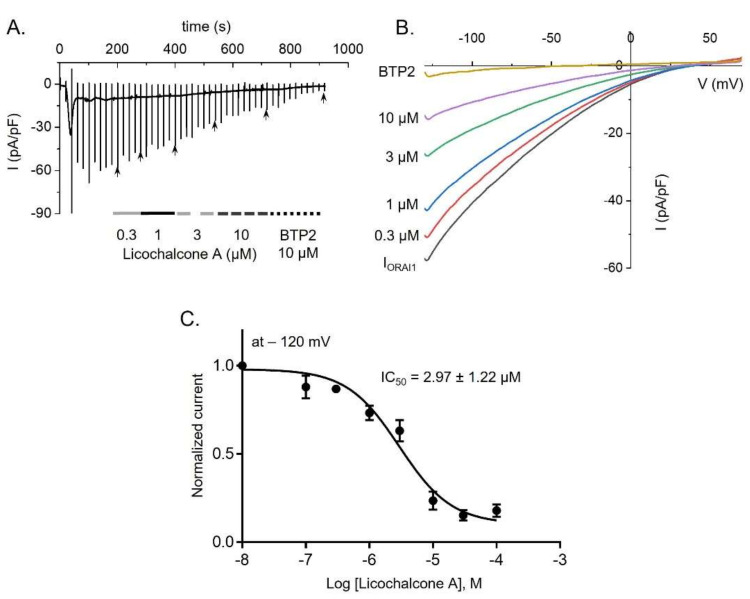
Inhibition of ORAI1 currents (I_ORAI1_) by licochalcone A in HEK293T cells overexpressing hORAI1 and hSTIM1. (**A**) A representative current trace indicates the suppressive effect of different concentrations of licochalcone A and BTP2 on I_ORAI1_. (**B**) Current (I)-voltage (V) relationship of I_ORAI1_ in the cells with or without treatment with licochalcone A. (**C**) Concentration-response image of licochalcone A suppression on I_ORAI1_, which was statistically analyzed at −120 mV. Data are presented as means ± SEM (*n* = 4–7 per dose).

To assess the suppressive effect of licochalcone A on ORAI1 currents, we recorded the currents on STIM1 and ORAI1-overexpressing HEK293T cells. Application of 20 nM IP_3_ and 20 mM BAPTA to the pipette solution, along with ramp pulses from −130 mV to +70 mV, robustly activated I_ORAI1_ to a steady peak, ensued by treatment with different concentrations of licochalcone A (0.1–100 µM) and 10 µM BTP2, which is known as potent inhibitor of ORAI1 channel (Figure 1A,B). Interestingly, licochalcone A inhibited I_ORAI1_ in a dose-dependent manner, which indicates a half maximal inhibitory concentration (IC_50_) of 2.97 ± 1.217 µM (Figure 1C).

### 2.2. Inhibitory Effect of Kv1.3 Currents by Licochalcone A in Jurkat T-Cells

**Figure 2:**
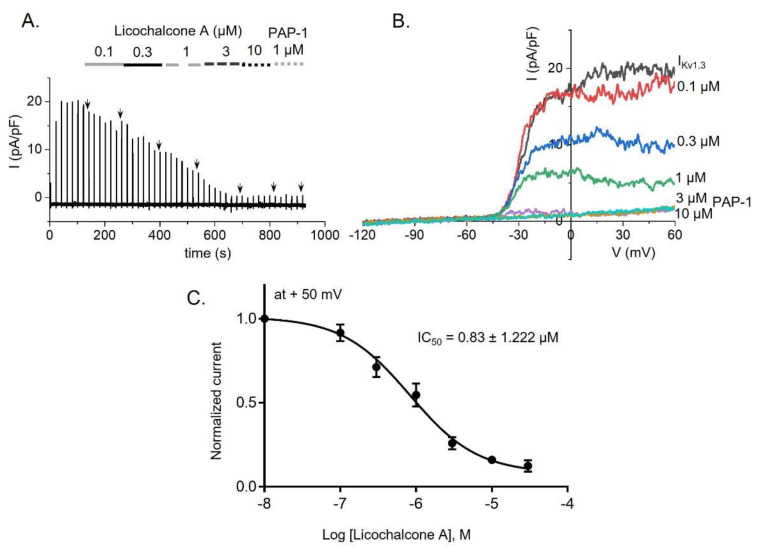
Inhibitory effect on Kv1.3 currents (I_Kv1.3_) by licochalcone A in Jurkat T-cells. (**A**) Whole-cell current recordings of I_Kv1.3_ after treatment with various concentrations of licochalcone A and PAP-1, a potent blocker of Kv1.3 channels. (**B**) Representative I-V relationships of I_Kv1.3_ in the cells after exposure to licochalcone A. (**C**) IC_50_ value obtained from a dose-dependent curve at +50 mV is indicated. Data are presented as means ± SEM (*n* = 5–11 per dose).

Kv1.3 channels have been found to be endogenously expressed in Jurkat T-cells [8,24,25]. Thus, we next studied the suppressive activity of licochalcone A on Kv1.3 currents in these human cells, which were activated by application of voltage-ramps from −120 mV to +60 mV (Figure 2A,B). As expected, while 1 µM PAP-1, a positive control for I_Kv1.3_, resulted in suppression by 79 ± 3.2%, Lichalcone A induced a dose-dependent reduction on the currents with an IC_50_ value of 0.83 ± 1.222 µM (Figure 2C).

### 2.3. Licochalcone A Inhibited KCa3.1 Currents in KCa3.1-Overexpressing HEK293 Cells

**Figure 3:**
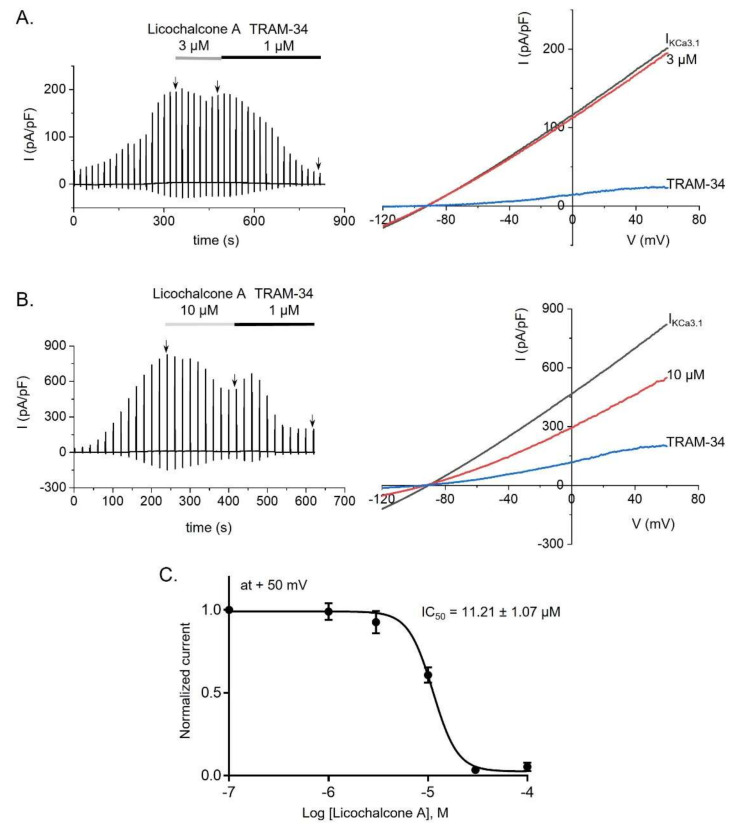
Licochalcone A inhibited KCa3.1 currents (I_KCa3.1_) in KCa3.1-overexpressing HEK293 cells. (**A**) A typical I_KCa3.1_ trace and I-V relationship demonstrates the effect of 3 µM licochalcone A on I_KCa3.1_ in KCa3.1-overexpressing HEK293 cells following treatment with TRAM-34 (1 µM), a selective blocking agent of the KCa3.1 channel. (**B**) A whole-cell trace and I-V relationships of I_KCa3.1_ in the cells treated with 10 µM licochalcone A and TRAM-34. (**C**) An IC_50_ value for licochalcone A suppression of I_KCa3.1_ in the cells. Data are presented as means ± SEM (*n* = 3–4 per dose).

In contrast to Kv1.3 channels, KCa3.1 is not expressed in Jurkat T-cells [26]. Hence, we employed KCa3.1-overexpressing HEK293 cells to examine whether licochalcone A could regulate KCa3.1 currents. The presence of free Ca^2+^ (1 μM) in the internal solution, along with the application of reversal potentials from +60 mV to −120 mV during 500 ms, activated I_KCa3.1_ to a peak amplitude, which was followed by treatment of licochalcone A at various concentrations and TRAM-34, a potent inhibitor of KCa3.1 currents (Figure 3A,B). Intriguingly, while 1 μM TRAM-34 was observed to suppress I_KCa3.1_ by 85.17 ± 2.25%, licochalcone A decreased the currents in a dose-dependent manner, with an IC_50_ of 11.21 ± 1.07 µM (Figure 3C).

### 2.4. Inhibition of Release of IL-2 and Proliferation of T-lymphocytes by Licochalcone A

**Figure 4:**
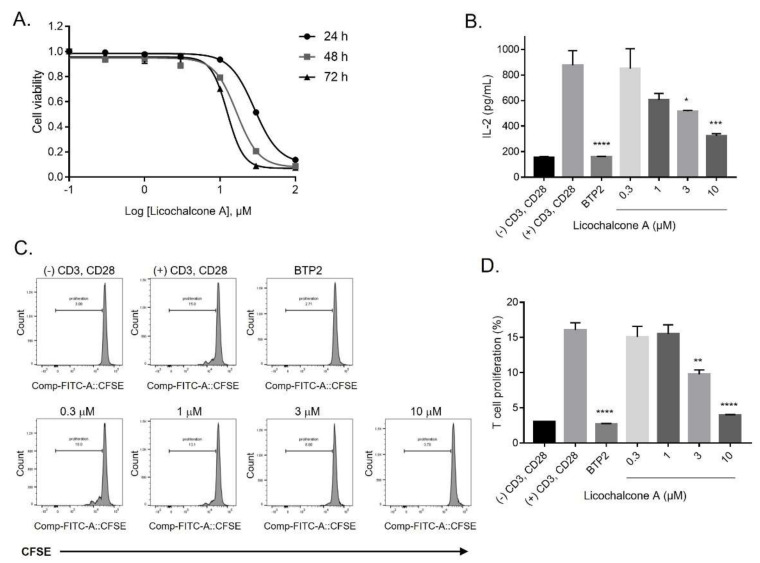
Licochalcone A suppressed IL-2 release and cell proliferation in T-lymphocytes. (**A**) Cytotoxicity curves of licochalcone A in Jurkat T-cells, measured after the cells were exposed to licochalcone A for 24 h, 48 h, and 72 h, using the CCK-8 assay. (**B**) Effect of licochalcone A on the release of IL-2 in CD3 and CD28-stimulated Jurkat T-cells was determined. Licochalcone A-untreated, stimulated cells were used as a control. Data are presented as means ± SEM (n = 3). (**C**) The purified human CD4^+^ T-cells were costimulated with CD3 and CD28 antibodies and stained with CFSE before treatment with 0.3, 1, 3, and 10 µM licochalcone A and BTP2. (**D**) The anti-proliferative efficacy of licochalcone A on a CD4^+^ T-cell population was quantitated. The untreated anti-CD3 and anti-CD28-costimulated cells were used as a control. Data are presented as means ± SEM (*n* = 3).

Before monitoring the immunosuppressive activity of licochalcone A via inhibition of IL-2 secretion and CD4^+^ T-cell proliferation, we examined licochalcone A cytotoxicity on Jurkat T-cells by performing a CCK-8 assay. As shown in Figure 4A, concentrations ≤ 10 µM of licochalcone A did not induce any changes on cell viability, except for 30 and 100 µM; these treatments showed approximately 50% cell death after different treatment periods (24 h, 48 h, and 72 h). Therefore, concentrations ≤10 μM of licochalcone A were selected for the following assay. Unexpectedly, a pretreatment of CD3 and CD28 antibody-stimulated Jurkat T-cells with 3 and 10 µM licochalcone A significantly decreased the production of IL-2 by 41.1 ± 0.64 and 63.3 ± 2.16%, respectively, compared with that of the stimulated cells without any treatments (Figure 4B).

In view of the anti-proliferative efficacy of licochalcone A, we pre-stimulated the CD4^+^ T-cells with both antibodies, anti-CD3 and anti-CD28, and pre-treated the cells with 0.3, 1, 3, and 10 µM licochalcone A and 10 µM BTP2 (Figure 4C). The results revealed that licochalcone A at 3 and 10 µM considerably suppressed T-cell growth by 39.2 ± 3.84 and 75.4 ± 0.68%, respectively, which showed a similar inhibition rate as that of 10 µM BTP2 (83.1 ± 0.49%) (Figure 4D).

## 3. Discussion

The anti-inflammatory activity of licochalcone A has been well defined in previous studies [10,14,16,17,18,19,20,21,22,23]. Specifically, licochalcone A has been reported to suppress the production of several proinflammatory mediators (IL-1β, PGE_2_, IL-6, NO, LTB_4_, COX-2, iNOS, and TNF-α) in different situations, including IL-1β-induced chondrocytes, N-formyl-MET-LEU-PHE, lipopolysaccharide (LPS), UVB-induced skin cell types (keratinocytes, granulocytes, fibroblasts, and dendritic cells), and the LPS-stimulated mouse mastitis model [16,17,19]. Additionally, licochalcone A decreased the secretion of anti-DNP IgE and DNP-HSA-induced β-hexosaminidase, a marker of RBL-2H3 mast cell degranulation, which is associated with allergic inflammatory responses [20]. Furthermore, licochalcone A obviously improved allergic inflammation through suppression of airway hyperresponsiveness, OVA-specific antibodies (IgG, IgG1, and IgE), and Th2 cytokines, which are comprised of IL-13, IL-5, and IL-4, while attenuating eosinophil infiltration and mucus hypersecretion in an OVA-induced asthma mouse model compared with normal mice [14,22]. However, its anti-inflammatory mechanism focusing on ion channels required further investigation.

ORAI1 and K^+^ channels (Kv1.3 and KCa3.1), extensively expressed in T-lymphocytes, are crucial targets in the treatment of a variety of immune diseases [8,9,27,28,29,30,31]. In particular, deletion of the ORAI1 gene in CD4^+^ T-cells has been reported to decrease the severe levels of experimental autoimmune encephalomyelitis and inflammation of the central nervous system via suppression of SOCE, as SOCE is involved in reducing the secretion of proinflammatory cytokines in Th1 and Th17 cells [28]. In addition, substantial amounts of inflammatory cytokines (INF-γ, IL-17, and TNF-α) from CD4^+^ T-cells were abundantly augmented due to upregulated expression of calcium-release-activated calcium channels in patients with rheumatoid arthritis [29]. As with ORAI1, experimental autoimmune encephalomyelitis was ameliorated by inhibition of Kv1.3 alone, or of both Kv1.3 and KCa3.1, in CD4^+^ T-lymphocytes [31]. The secretion of several proinflammatory cytokines (INF-γ, IL-17, and TNF-α) was reduced by blockage of Kv1.3 and KCa3.1 in T-lymphocytes from patients with ulcerative colitis [30]. Hence, in the current study, we aimed to demonstrate the anti-inflammatory effect of licochalcone A via modulation of all three channels. Our results revealed that licochalcone A has a concentration-dependent inhibitory influence on ORAI1, Kv1.3, and KCa3.1 with IC_50_ values of 2.97 ± 1.217, 0.83 ± 1.222, and 11.21 ± 1.07 µM, respectively (Figure 1, Figure 2 and Figure 3).

However, due to the multiple effects of licochalcone A on the ion channels, additional studies are required to determine whether the compound targets only specific ion channels that are important for the immune response. Although the results described here are part of a preliminary analysis, we analyzed the effect of licochalcone A on other ion channels, including TRPV3 and TMEM16A (ANO1, a calcium activated Cl-channel). The results shown in Supplementary Figure S1 indicate that 10 µM licochalcone A did not affect the ion channel activities of TRPV3 and TMEM16A. These results indicate that Licochalcone A is not a non-selective ion channel inhibitor. Additional studies are required to determine whether Licochalcone A targets ion channels that are primarily expressed in immune cells.

The activation of these channels promotes ([Ca^2+^]_i_) following the engagement of human T-cells with antigen-presenting cells [8]. Indeed, licochalcone A was previously reported to dose-dependently reduce increases in ionomycine-induced ([Ca^2+^]_i_) in human polymorphonuclear neutrophils [32]. These results confirmed that licochalcone A exerted its inhibitory effect on ([Ca^2+^]_i_) via modulation of all three channels in the Jurkat T-cells.

Calcium signaling, as well as the ORAI1 and K^+^ channels, contributes to the secretion of inflammatory cytokines, including IL-2 [1,6,9,33]. Thus, we investigated the influence of licochalcone A on the release of IL-2 in the Jurkat T-cells. To ensure its effect on IL-2 was not caused by its cytotoxicity, we examined the toxicity of licochalcone A in the Jurkat T-cells using the CCK-8 assay. Our results indicate that licochalcone A at concentrations ≤10 μM insignificantly affect cell viability, while exposure of the cells to 30 and 100 μM was associated with a high rate of cell death (Figure 4A). Consistent with previous reports, licochalcone A demonstrated its negligible cytotoxicity in several cell lines, including RBL-2H3, HELF, SH-SY5Y, EGS-1, BEAS-2B, and RAW 264.7 at doses ≤ 10 μM [10,20,23,34], while treatment of 20, 40, and 60 μM of licochalcone A showed insignificant toxicity in the MC3T3-E1, a normal osteoblast cell line [35]. These results further confirmed that licochalcone A is safe to administer in experiments using cell lines at doses ≤ 10 μM. Thus, these doses were then chosen to evaluate the effect of licochalcone A on the release of IL-2 in the CD3 and CD28 antibody-induced Jurkat T-cells. Interestingly, licochalcone A resulted in a significant suppression of the release of IL-2 (Figure 4B). Collectively, these results suggest that licochalcone A might play an essential role in immunomodulation by blocking ORAI1 and K^+^ channels (Kv1.3 and KCa3.1) in T-lymphocytes.

The Ca^2+^ signaling mediating all three channels has been well defined to play a vital role in T-cell proliferation [3]. Here, we demonstrated that licochalcone A reduced the proliferation of anti-CD3 and anti-CD28-induced human naïve T-lymphocytes (Figure 4C,D). Our results corroborate those of previous studies, which indicated that licochalcone A has an anti-proliferative effect on a variety of cancer cell lines [36,37,38,39,40,41]. More intriguingly, licochalcone A suppressed the proliferation of HCT116 cells, a human colon cancer cell line, while promoting T-cell activity, which increased the killing of tumor cells by T-cells in a coculture model of both cells [36]. Similarly, the population of human gastric cancer cells, known as BGC cells, was also inhibited by the application of licochalcone A, which was not observed in normal human gastric cells [38]. These results demonstrate that licochalcone A might be a potential and safe anti-tumor immunotherapeutic agent without influencing host cells.

## 4. Materials and Methods

### 4.1. Reagents

Licochalcone A, TRAM-34, and PAP-1 were purchased from Sigma-Aldrich (St. Louis, MO, USA). IP_3_ was purchased from Merck Millipore (Billerica, MA, USA), and BTP2 was obtained from Tocris (Bristol, UK). All chemicals, except IP_3_, were dissolved in dimethyl sulfoxide, which was prepared in distilled water.

### 4.2. Cell Line and Cell Culture

HEK293T, RBL-2H3, Jurkat T-cells, and a cell line overexpressing KCa3.1 were obtained from ATCC (Manassas, VA, USA). All cells were cultured according to a previously described method [42,43]. In brief, the HEK293T and KCa3.1-overexpressing cell lines were maintained in DMEM (Welgene, Gyeongsan, Korea), whereas the Jurkat T-cells were maintained in RPMI1640 (Thermo Fisher Scientific, Waltham, MA, USA); these media were supplemented with a final concentration of 10% FBS (Welgene, Gyeongsan, Korea) and 1% penicillin/streptomycin (Hyclone, Marlborough, MA, USA).

### 4.3. Transient Transfection

After the HEK293T was seeded for 24 h (50~70% confluency), hORAI1, hSTIM1, and green fluorescence vectors (Origene Technologies, Rockville, MD, USA) were transiently co-transfected into the cells using TurboFect (Thermo Scientific, Waltham, MA, USA) according to the manufacturer’s guidelines. After a 24–36 h transfection, green fluorescence-enhanced cells were used for recording the ORAI1 currents (I_ORAI1_).

### 4.4. Electrophysiological Experimentation

For whole-cell patch clamp measurements, we used a sophisticated system including a flaming/brown micropipette puller apparatus (Model P-97, Shutter Instrument, Novato, CA, USA) to obtain the glass pipettes by pulling the borosilicate capillary (World Precision Instruments, Inc. Sarasota, FL, USA) and fire-polishing to an electrical resistance of 2–3 (MΩ) (Narishige, East Meadow, NY, USA). In addition, a digitizer of 10 kHz and a low pass-filter of 5 kHz were established using a Digidata 1440A (Molecular Devices) and an Axopatch 700B (Molecular Devices, Sunnyvale, CA, USA), respectively. Origin 8.0 software (MicroCal ITC, Northampton, MA, USA) and a Clampfit 10.4 (Molecular Devices) were also used. ORAI1 currents were recorded on the hORAI1 and hSTIM1-cotransfecting HEK293T using the bath (external) solution, which contained 10 mM HEPES, 135 mM NaCl, 10 mM CaCl_2_, 5 mM D-glucose, 1 mM MgCl_2_, and 3.6 mM KCl (pH 7.4 with NaOH). The internal (pipette) solution contained 20 mM BAPTA, 130 mM Cs-glutamate, 3 mM Mg-ATP, 1 mM MgCl_2_, 20 mM HEPES, and 0.002 mM sodium pyruvate (pH 7.2 with CsOH). ORAI1 channels were activated by the application of ramp pulses from −130 to +70 mV within 100 ms, with the cell membrane holding at +10 mV. 

For the KCa3.1 current (I_KCa3.1_) recordings, the external solution contained 5 mM glucose, 145 mM NaCl, 1.3 mM CaCl_2_, 10 mM HEPES, 3.6 mM KCl, and 1 mM MgCl_2_ (pH 7.4 with NaOH). The pipette solution with 1 µM free Ca^2+^ contained 0.5 mM MgCl_2_, 5 mM NaCl, 10 mM HEPES, 2 mM Mg-ATP, 140 mM KCl, 4.37 mM CaCl_2_, and 5 mM EGTA (pH 7.2 with KOH). The free ([Ca^2+^]_i_) of the pipette solution was determined using WebMaxC (https://somapp.ucdmc.ucdavis.edu/pharmacology/bers/maxchelator/webmaxc/webmaxcS.htm (accessed on 15 January 2021). The current-voltage relationships were constructed by setting ramp pulses from +60 to −120 mV over 500 ms. The membrane of the KCa3.1-overexpressing cell line was held at −90 mV. 

The Jurkat T-cells were used for measuring the whole-cell Kv1.3 currents (I_Kv1.3_). The bath solution for recording the I_Kv1.3_ contained the same compositions as that for measuring the I_KCa3.1_, whereas the pipette solution with the free Ca^2+^ contained 5 mM NaCl, 10 mM HEPES, 2 mM Mg-ATP, 140 mM KCl, 5 mM EGTA, and 0.5 mM MgCl_2_ (pH 7.2 with KOH). The I_Kv1.3_ was elicited by application of ramp pulses from −120 to +60 mV for 500 ms, with the cell membrane holding at −60 mV.

### 4.5. Human CD4^+^ T-Lymphocyte Assay

This assay was approved by the healthy donors and the Institutional Review Board (IRB), Dongguk University College of Medicine, South Korea (2017-07-003 IRB). A Ficoll-Paque Plus medium (GE Healthcare, Chicago, IL, USA) and a CD4^+^ T-cell isolation kit (Miltenyi Biotec, Bergisch Gladbach, Germany) were used for the isolation of T-cells. After isolation, the T-lymphocytes were washed with DPBS followed by resuspension at a density of approximately 10^5^–10^6^ cells/mL. Subsequently, the cells were labeled with carboxyfluorescein succinimidyl ester (CFSE) (Thermo Fisher Scientific) before incubation at 25 °C for an additional 10 min. The incubation was interrupted by complete media in cold conditions, and the cells were placed on ice for an additional 5 min. For a co-stimulatory reaction, the mixture of anti-CD28 (2 μL/mL) and CFSE-tagged cells (2 × 10^5^ cells/well) were added to anti-CD3 (5 μL/mL)-precoated wells (96-well plates), ensued by a 72 h incubation. The T-cell growth was determined by flow cytometry (LSRFortessa, BD bioscience, NJ, USA).

### 4.6. Cell Cytotoxicity

The cytotoxicity of licochalcone A on the Jurkat T-cells was assessed by the CCK-8 assay (Dojindo Laboratories, Japan). A good passage of the Jurkat T-cells was selected, seeded at a density of 2 × 10^4^ cells/well (100 µL cell suspension/well in 96-well plates), treated with 0.3–100 µM licochalcone A, and ensued by different incubation periods (24 h, 48 h, and 72 h). Afterwards, the cells were incubated with fresh CCK-8 (10 µL) in darkness for 2 h. The plates were centrifuged at 1500 rpm for 1 min 30 s at 25 °C before absorbance measurements at 450 nm.

### 4.7. Measurement of IL-2 Secretion

Precoating with 50 µL anti-CD3 per well (5 μg/mL; Peprotech, Rocky Hill, NJ, USA) in a 96-well plate was performed prior to incubation for 3 h at 37 °C. Then, the precoated wells were washed three times with DPBS to remove the excess anti-CD3. As treatment, different concentrations of licochalcone A were added to a mixture of anti-CD28 (2 μg/mL; Peprotech) and Jurkat T-cells (2 × 10^5^ cells/well); the mixture was subsequently plated in each well, followed by incubation for 48 h. To determine the levels of IL-2, the supernatants of the untreated and treated cells were collected and measured using a human IL-2 ELISA kit (Peprotech).

### 4.8. Data Analysis

Data are represented as the mean ± standard error of the mean (SEM). Additionally, comparisons of various groups were performed using a one-way ANOVA followed by a Bonferroni correction. Variants were statistically significant at *, **, ***, and **** corresponding to P values of <0.05, <0.01, <0.001, and <0.0001, respectively. The statistical analysis software GraphPad (GraphPad, La Jolla, CA, USA) was used to perform all analyses.

## 5. Conclusions

In summary, the current study demonstrates that licochalcone A is an inhibitor of the ORAI1, Kv1.3, and KCa3.1 channels. Further, licochalcone A shows remarkable suppression of IL-2 release and proliferation in T-lymphocytes, cells that are vital in the modulation of inflammation. Collectively, our results provide evidence that licochalcone A might be a promising and potent anti-inflammatory agent for the treatment and prevention of autoimmune diseases.

## Data Availability

The data that support the findings of this study are available from the corresponding author upon reasonable request.

## Supplementary Materials

The following are available online at https://www.mdpi.com/article/10.3390/ijms221910847/s1, Figure S1: The effect of Licochalcone A on TMEM16A and TRPV3 currents in HEK293T cells overexpressing the respective channels.
