# Supplementary material for: Anti-Inflammatory Effect of Licochalcone A via Regulation of ORAI1 and K+ Channels in T-Lymphocytes
Source: Int J Mol Sci. 2021 Oct 7;22(19):10847. doi: 10.3390/ijms221910847 (PMC8509259; doi:10.3390/ijms221910847)
Supplement: Supplementary file 1 [file ijms-22-10847-s001.zip › ijms-1386458-supplementary.pdf]

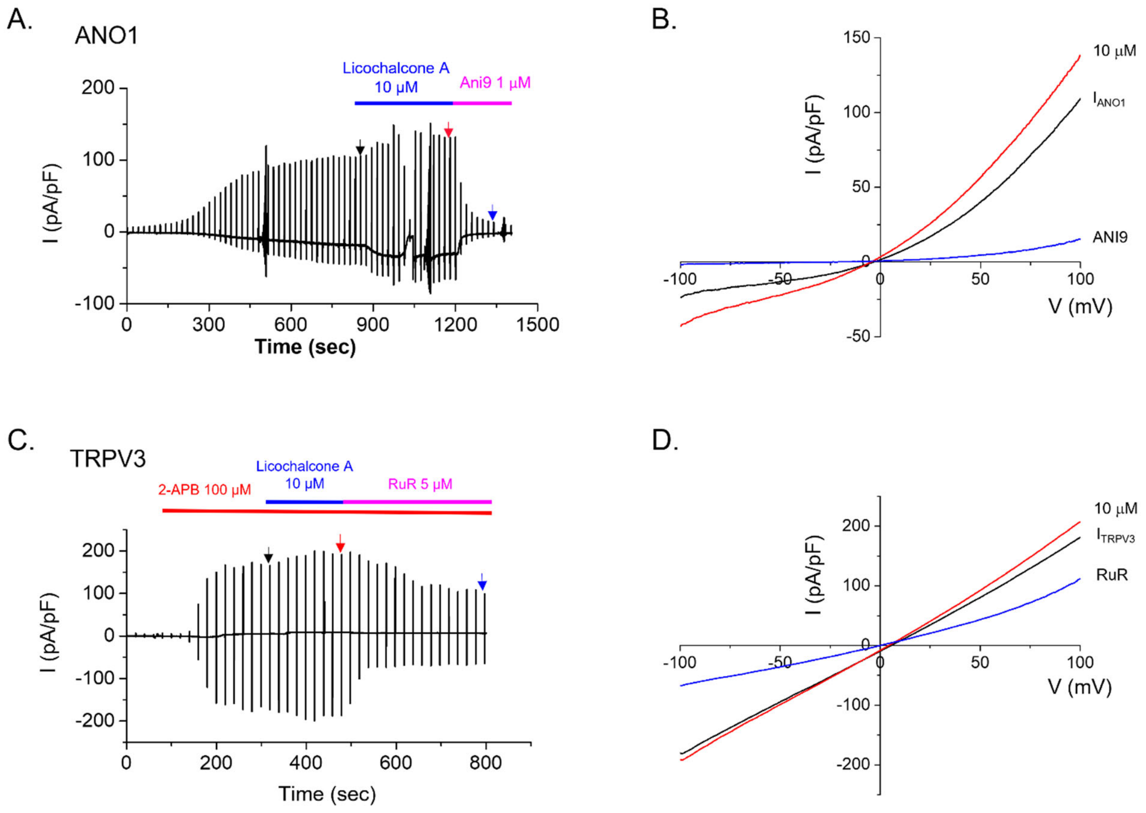

Figure S1. The effect of Licochalcone A on TMEM16A and TRPV3 currents in HEK293T cells overexpressing the respective channels**.** (A, B) Representative induced whole-cell current (I_{ANO1}) trace (A) and its I–V relationships for TMEM16A. (B) No effect of 10 µM licochalcone A was observed on I_{ANO1}. (C, D) A typical whole-cell TRPV3 current (I_{TRPV3}) trace activated using 100 µM 2-APB (C) and the related I–V curves (D) in the presence of 10 µM licochalcone A. Ani9 and ruthenium red were used as specific inhibitors for ANO1 and TRPV3, respectively.
